# Systematic analysis of IL-6 as a predictive biomarker and desensitizer of immunotherapy responses in patients with non-small cell lung cancer

**DOI:** 10.1186/s12916-022-02356-7

**Published:** 2022-05-13

**Authors:** Chengming Liu, Lu Yang, Haiyan Xu, Sufei Zheng, Zhanyu Wang, Sihui Wang, Yaning Yang, Shuyang Zhang, Xiaoli Feng, Nan Sun, Yan Wang, Jie He

**Affiliations:** 1 Department of Thoracic Surgery, National Cancer Center/National Clinical Research Center for Cancer/Cancer Hospital, Chinese Academy of Medical Sciences and Peking Union Medical College, Beijing, 100021 China; 2 State Key Laboratory of Molecular Oncology, National Cancer Center/National Clinical Research Center for Cancer/Cancer Hospital, Chinese Academy of Medical Sciences and Peking Union Medical College, Beijing, 100021 China; 3 Department of Medical Oncology and Radiation Sickness, Peking University Third Hospital, Beijing, 100191 China; 4 Department of Medical Oncology, National Cancer Center/National Clinical Research Center for Cancer/Cancer Hospital, Chinese Academy of Medical Sciences and Peking Union Medical College, Beijing, 100021 China; 5 Department of Comprehensive Oncology, National Cancer Center/ National Clinical Research Center for Cancer/Cancer Hospital, Chinese Academy of Medical Sciences and Peking Union Medical College, Beijing, 100021 China; 6 Department of Pathology, National Cancer Center/National Clinical Research Center for Cancer/Cancer Hospital, Chinese Academy of Medical Sciences and Peking Union Medical College, Beijing, 100021 China

**Keywords:** NSCLC, Immune checkpoint inhibitors, IL-6, Clinical response, Resistance

## Abstract

**Background:**

Cytokines have been reported to alter the response to immune checkpoint inhibitors (ICIs) in patients with the tumor in accordance with their plasma concentrations. Here, we aimed to identify the key cytokines which influenced the responses and stimulated resistance to ICIs and tried to improve immunological response and develop novel clinical treatments in non-small cell lung cancer (NSCLC).

**Methods:**

The promising predictive cytokines were analyzed via the multi-analyte flow assay. Next, we explored the correlation baseline level of plasma cytokines and clinical outcomes in 45 NSCLC patients treated with ICIs. The mechanism of the potential candidate cytokine in predicting response and inducing resistance to ICIs was then investigated.

**Results:**

We found NSCLC with a low baseline concentration of IL-6 in plasma specimens or tumor tissues could derive more benefit from ICIs based on the patient cohort. Further analyses revealed that a favorable relationship between PD-L1 and IL-6 expression was seen in NSCLC specimens. Results in vitro showed that PD-L1 expression in the tumor was enhanced by IL-6 via the JAK1/Stat3 pathway, which induced immune evasion. Notably, an adverse correlation was found between IL-6 levels and CD8^+^ T cells. And a positive association between IL-6 levels and myeloid-derived suppressor cells, M2 macrophages and regulator T cells was also seen in tumor samples, which may result in an inferior response to ICIs. Results of murine models of NSCLC suggested that the dual blockade of IL-6 and PD-L1 attenuated tumor growth. Further analyses detected that the inhibitor of IL-6 stimulated the infiltration of CD8^+^ T cells and yielded the inflammatory phenotype.

**Conclusions:**

This study elucidated the role of baseline IL-6 levels in predicting the responses and promoting resistance to immunotherapy in patients with NSCLC. Our results indicated that the treatment targeting IL-6 may be beneficial for ICIs in NSCLC.

**Graphical Abstract:**

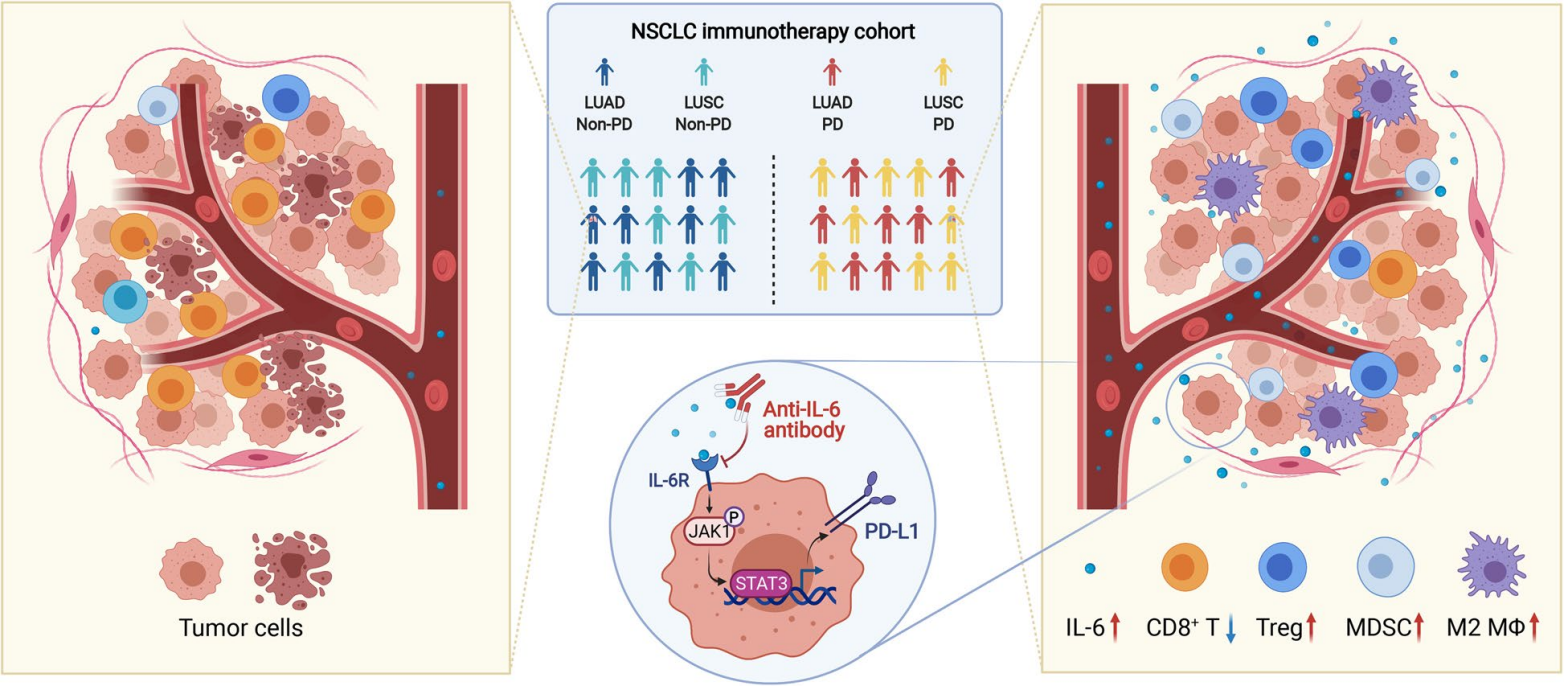

**Supplementary Information:**

The online version contains supplementary material available at 10.1186/s12916-022-02356-7.

## Background

Non-small cell lung cancer (NSCLC) represents about 80% of patients with lung cancer which is associated with the highest cancer-related mortality worldwide [1, 2]. Compared with conventional therapy, immune checkpoint inhibitors (ICIs) have been reported to enhance responses to treatment and extend overall survival (OS) in patients with diagnosed advanced NSCLC [3, 4]. ICIs block certain immune checkpoints, alleviate immunosuppression, and boost immune responses in patients with cancer [5, 6]. However, the objective response rate of ICIs against PD-1/PD-L1 is only 19–20% [3, 4]. Therefore, predictive biomarkers are needed to explore and guide the selection of patients in whom ICIs may be effective.

Currently, PD-L1 expression in tumor tissues is the most widely recognized biomarker to guide the selection of targeted immunotherapy in patients with advanced NSCLC. Nevertheless, the predictive performance of such an approach is modest in clinical practice [7, 8]. In addition, when tumor specimens cannot be obtained due to infaust tumor location, the therapeutic options would be limited due to the unavailable of the PD-L1 expression. However, plasma can be obtained instead in such situation, with the advantages of easy access for follow-up analyses. Hence, it is important to identify effective plasma biomarkers to inform anticancer treatments involving ICIs.

Cytokines are soluble proteins that are secreted by immune cells which include interferons (IFNs), chemokines, interleukins, colony-stimulating factors, tumor necrosis factors, and growth factors [9–11]. Elevated levels of cytokines can be detected in patients exhibiting immune responses to inflammation and cancer, while homeostatic concentrations are typically low or undetectable [12–14]. Cytokines, as important regulators of immune activity, recruit immune cells to the tumor immune microenvironment (TIME) and promote the expression of certain immune checkpoint molecules in the procession of antitumor activities [15]. Thus, these circulating cytokines could be potentially predictive biomarkers for responses to ICIs. A previous study reported that low baseline plasma levels of granzyme B correlated with an unsatisfactory response to immunotherapy in NSCLC patients who received nivolumab [16]. Another study demonstrated that baseline plasma IL-6 levels played a negative role in predicting the efficacy of immunotherapy in NSCLC [17]. However, little systematic research has analyzed the role of circulating cytokines in predicting the response to therapies and survival benefit from ICIs in patients with NSCLC.

Changes in cytokine levels have been demonstrated in the previous studies as a result of immunotherapy which can regulate the proliferation and differentiation of immune cells, while influencing the proliferation and metastasis of cancer cells in the TIME as well [18, 19]. Notably, cytokine therapy to promote immune activity in patients with cancer plays an important role in current clinical cancer research. For example, IFN-α has been approved as an adjuvant treatment for melanoma. High-dose IL-2 has been approved as a treatment for both melanoma and metastatic renal cell cancer. In addition, the targeted inhibition of IL-6 has been reported to promote T cell infiltration and enhance the efficacy of ICIs to treat colorectal and pancreatic cancers in recent studies [20, 21]. Therefore, the role of cytokines in the treatment of NSCLC should be further investigated.

In the present study, we explored the association between baseline levels of plasma cytokines and clinical benefits observed in patients with NSCLC treated with ICIs using a multi-analyte flow assay and identified promising predictive cytokine biomarkers. Furthermore, the mechanism of candidate cytokines in predicting the responses and inducing resistance to ICIs was analyzed in this study. Importantly, we explored these candidate cytokines as potential targets of combination immunotherapies to inform new clinical treatments for patients with NSCLC.

## Methods

### Patient enrollment and response evaluation

A total of 45 patients diagnosed with NSCLC and treated with anti-PD-1 inhibitors from January 2018 to December 2019 at the Cancer Hospital and Institute, Chinese Academy of Medical Sciences (CICAMS, Beijing, China) were included in the present study. Enrolled patients received immunotherapy once every 2 or 3 weeks (nivolumab was administered once every 2 weeks, other anti-PD-1 drugs were given once every 3 weeks). Radiologic assessments, including computed tomography, were conducted every 6 weeks. If necessary, head magnetic resonance imaging was simultaneously performed. The tumor response was assessed according to the Response Evaluation Criteria in Solid Tumors (version 1.1) and was categorized as complete response (CR), partial response (PR), stable disease (SD), or progressive disease (PD). Progression-free survival (PFS) and OS were defined as the time from the initiation of ICIs administration to the time of PD and from the beginning of ICIs to death, respectively. The last follow-up assessment was performed on January 2, 2021. The Ethics Committee of CICAMS approved this study (approval number 19/147-1925). Clinical characteristics of these NSCLC patients are described in Additional file 1: Table S1.

### Sample collection and cytokine assessment

Blood samples were collected in an EDTA tube before the administration of anti-PD-1 immunotherapy. All samples were processed within 2 h of collection, and centrifuged at 3000 rpm for 10 min at 4 °C. The upper plasma fraction was stored at − 80 °C until assayed. Plasma samples were obtained from NSCLC patients to perform the cytokine assessment. The levels of secreted cytokines, including IL-17A, TNF-α, IL-2, IL-10, IL-4, IFH-γ, IL-6, and granzyme B, were quantified using a custom LEGENDplex™ Human Multi-Analyte Flow Assay kit (Cat# 740267; BioLegend). Data were gained via the Flow Cytometer (BD Biosciences) with the manufacturer’s instructions. Furthermore, the human IL-6 enzyme-linked immunosorbent assay (ELISA) kit (Cat# ab46042; Abcam) was applied to measure the levels of IL-6.

### Specimen collection and immunohistochemistry (IHC)

We collected tissue specimens and archived the samples via formalin fixation paraffin embedding (FFPE) before the initiation of anti-PD-1 immunotherapy. FFPE samples from 25 patients with NSCLC were examined for protein levels of IL-6. IHC was used to assess the expression of IL-6 with an anti-IL-6 monoclonal antibody (mAb; Cat# 12153; CST, USA). The staining score of IL-6 in every tissue sample was calculated using the following formula: IHC score of IL-6 = staining intensity × percentage of positive tumor cells × 100. The staining intensity score was divided into four grades: no color was zero (negative), light yellow was 1 (weakly positive), yellow was 2 (moderately positive), and brownish yellow was 3 (strongly positive). Ten fields were randomly selected under a high-power microscope (× 400). The average value was taken to calculate the percentage of tumor cells that stain positively compared to all tumor cells in view. All slides were assessed by two pathologists independently based on the evaluation criteria of the previously published method [22]. These pathologists were blinded to the patients’ clinical characteristics.

### Clinical cohort and TIME analysis

Tumor samples were resected from 196 patients with NSCLC, including 137 patients diagnosed with lung adenocarcinoma (LUAD) and 59 with lung squamous carcinoma (LUSC), between January 2012 and December 2016. These specimens were analyzed for IL-6 expression, PD-L1 expression, and numbers of CD8^+^ T cells, myeloid-derived suppressor cells (MDSCs), M2 macrophages, and regulatory T cells (Treg cells). Using IHC, tumor sections were stained for PD-L1 (pre-diluted anti-human PD-L1 mAb, clone SP263, Cat# 740-4907; Ventana, USA) using an automated Ventana Benchmark XT instrument, CD163 (pre-diluted anti-human CD163 mAb, Cat# ZM-0428; Zsbio Tech, China), Foxp3 (anti-human Foxp3 mAb, Cat# ab20034; Abcam, UK), and CD8 (pre-diluted anti-human CD8 mAb, Cat# ZA-0508; Zsbio Tech, China) [23, 24]. MDSCs were analyzed for CD11b expression (anti-human CD11b mAb, Cat# ab52478; Abcam, UK) and CD33 expression (anti-human CD33 mAb, Cat# ab30371; Abcam, UK) by double immunofluorescence [25]. The PD-L1 tumor proportion score and the proportion of immune cells were assessed according to the evaluation criteria of the previously published approach [26].

### Cell lines and reagents

Immortalized human bronchial epithelial cells (Beas-2B cells) were cultured in BEGM media (Cat# CC-3170; Lonza, Switzerland) containing 10% fetal bovine serum (FBS, Cat# 35-081-CV, Corning, USA). Human LUAD cell lines (H358, A549, PC-9, and H1975) were cultured in RPMI-1640 media (Cat# 10-040-CV, Corning, USA) containing 10% FBS and an antibiotic mixture (100 U/mL penicillin and 100 μg/mL streptomycin, Cat# 15140-122, Gibco, USA). Human LUSC cell lines (H226 and H1703) were cultured in RPMI-1640 media containing 10% FBS and an antibiotic mixture. Mouse LA795 cells (clones from the lung neoplasm of a 615 mouse with LUAD) were cultured in RPMI-1640 media containing 10% FBS and an antibiotic mixture. Mouse KLN205 cells (clones from the lung neoplasm of a DBA-2 J mouse with LUSC) were cultured in DMEM media (Cat# 10-013-CVR, Corning, USA) containing 10% FBS and an antibiotic mixture. Beas-2B, H358, A549, H1975, H226, and KLN205 were purchased from American Type Culture Collection. PC-9, H1703, and LA795 were purchased from Cell Resource Center in the Institute of Basic Medical Sciences, Chinese Academy of Medical Sciences.

The reagents used in this study included human recombinant IL-6 (Cat# 200-06-20, PeproTech, USA), human recombinant IL-2 (Cat# 200-02-50, PeproTech, USA), and JAK1/2 inhibitor (ruxolitinib, Cat# S1378, Selleck, China). The antibodies for western blot analysis used in this study are listed as follows: IL-6 (Cat# 12153; CST, USA), PD-L1 (Cat# 13684 T; CST, USA), JAK1 (Cat# 3344; CST, USA), phospho-JAK1 (Cat# 3331; CST, USA), Stat3 (Cat# 9139; CST, USA), phospho-Stat3 (Cat# 4113; CST, USA), and GAPDH (Cat# ab8245, Abcam, UK).

### Stable lentiviral IL-6 overexpression

Full-length IL-6 cDNA was ligated into the pHS-BVC-LW334 vector. According to the manufacturer’s guidelines, HEK-293 T cells were co-transfected with the IL-6 vector and packaging plasmid (pLP1, pLP2, and pLP/VSVG) using Lipofectamine 3000 (Cat# L3000015, Invitrogen, USA) to produce lentivirus carrying the IL-6 gene. A549 and H1703 cells were then infected with harvested lentiviruses and exposed to 5 μg/mL polybrene (Cat# P4505, Sigma-Aldrich, USA) and selected for mCherry expression using flow cytometry. The expression levels of IL-6 in the infected cells were confirmed by western blot analysis after selection.

### In vitro tumoricidal activity assays

Human peripheral blood mononuclear cells (PBMCs) were extracted by Ficoll centrifugation (Cat# 17-1440-02, GE Healthcare, USA) from peripheral blood provided by healthy donors. We then isolated CD8^+^ T cells from PBMCs using the human CD8^+^ T Cell Isolation kit (Cat# 130-096-495, Miltenyi Biotec, Germany). Isolated CD8^+^ T cells were activated by the addition of human anti-CD3/CD28 Dynabeads (Cat# 40203D, Thermo Fisher Scientific, USA) for 3 days and cultured in RPMI-1640 media containing 10% FBS, an antibiotic mixture, and 200 U/mL IL-2. After adherent overnight, control or IL-6-overexpressing A549 and H1703 cells were incubated with activated CD8^+^ T cells for 48 h, and exposed to either the isotype control or durvalumab (1500 μg/mL). In addition, after adherent overnight, A549 and H1703 cells were incubated with activated CD8^+^ T cells for 48 h with or without exposure to the IL-6 (20 ng/mL) isotype control or durvalumab (1,500 μg/mL). The ratio of CD8^+^ T cells to cancer cells was 5:1. After the removal of T cells and cellular debris, living cancer cells were then quantified using the Cell Counting Kit-8 (Cat# CK04, DOJINDO, Japan). The tumoricidal activity was calculated using the following formula: killing activity (%) = [1 − (optical density value of experimental group − optical density value of T cells group)/optical density value of tumor cells group] × 100%.

### In vivo mouse experiments

Female 615 mice (SPF, 5–6 weeks old) were purchased from the Institute of Hematology, Chinese Academy of Medical School (Tianjin, China). Male DBA-2 J mice (SPF, 5–6 weeks old) were purchased from Huafukang Bioscience Co., Ltd. (Beijing, China). The mice were housed under standard conditions in the animal care facility at the Center of Experimental Animals of CICAMS. The temperature and humidity were maintained at 26 °C–28 °C and 60 ± 5%, respectively. All procedures were approved by the Animal Care and Use Committee of CICAMS. As shown in Figs. 4A and 5A, to establish the LUAD and LUSC mouse models to study the respective immune systems, 615 mice and DBA-2 J mice were subcutaneously implanted with murine LA795-derived xenografts and KLN205-derived xenografts, respectively. We measured tumor size using a caliper every three days and calculated the tumor volume based on the formula *V* = *L* × *W* ^2^/2 (*V*: tumor volume; *L*: tumor length; *W*: tumor width). The mice were randomly placed into five groups with five mice/group and given different treatments when the maximum tumor diameter reached 7 mm. The treatment regimens involved anti-PD-L1 mAb (10 mg/kg three times a week, BioXCell, USA), anti-IL-6 mAb (10 mg/kg three times a week, BioXCell, USA), a combination of anti-PD-L1 and anti-IL-6 drugs, or a combination of an anti-PD-L1 drug and paclitaxel (20 mg/kg twice a week, CSPC Inc., China) as described in Figs. 4A and 5A. The endpoints were defined when the maximum tumor diameter reached 20 mm, the weight loss was greater than 2 g, or death. All mice were sacrificed by carbon dioxide asphyxiation to harvest tumors.

Harvested tumors were fixed in formalin, embedded in paraffin, and sectioned (4 μm). Using IHC, tumor sections were stained for CD8 (anti-mouse CD8 antibody, Cat# 98941; CST, USA), CD163 (anti-mouse CD163 antibody, Cat# ab182422; Abcam, UK), Foxp3 (anti-mouse Foxp3 antibody, Cat# ab215206; Abcam, UK), and PD-L1 (anti-mouse PD-L1 antibody, Cat# ab238697; Abcam, UK). MDSCs were stained for CD11b (anti-mouse CD11b antibody, Cat# ab133357; Abcam, UK) and Ly6G (anti-mouse Ly6G antibody, Cat# GB11229; Servicebio, China) using double immunofluorescence analysis. The staining score of PD-L1 in mouse samples was calculated using the following formula: IHC score of PD-L1 = staining intensity× percentage of positive tumor cells × 100. The proportion of immune cells was assessed according to the evaluation criteria of the previously published approach [26]. Ten fields were randomly selected under a high-power microscope (× 400) in subcutaneous tumor nodule per mouse. The average value was taken to calculate the percentage of immune cells that stain positively compared to all immune cells in view. All slides were assessed by two experienced pathologists blinded to the clinical parameters.

### Statistical analysis

Data analysis was conducted using R software (version 3.6.0), SPSS (version 22.0; IBM, New York, USA), and GraphPad Prism software (version 8.0, Graph Pad, San Diego, CA, USA). Differences between independent variables were evaluated using the Kruskal-Wallis H test or Mann-Whitney *U* test. Fisher’s exact test was used to analyze categorical variables. Correlation coefficients were obtained using Pearson correlation analysis. Survival was assessed with the log-rank test and Kaplan-Meier analysis. Furthermore, Cox regression was conducted for univariate and multivariate analyses of prognosis. Notably, all factors with a *P* value less than 0.15 in the log-rank test were analyzed in the multivariate Cox regression analysis. All statistical analyses were double-sided, and statistical significance was considered as *P* values less than 0.05.

## Results

### NSCLC patients with low baseline levels of IL-6 in plasma specimens and tumor tissues could derive more benefit from ICIs

A total of 45 patients with advanced NSCLC receiving ICIs monotherapy were included in this study (Additional file 1: Table S1). Twenty patients (44.4%) were diagnosed with LUAD, and 25 (55.6%) were diagnosed with LUSC. All patients received anti-PD-1 antibodies. Seven patients (15.6%) were determined to have PR, 20 patients to have SD (44.4%), and 18 to have PD (40%). The median PFS was 4.17 months (95% CI 2.49–5.85), and the median OS was 24.07 months (95% CI 13.32–34.81). The median follow-up length was 18.30 months (95% CI 15.26–21.34).

**Fig. 1:**
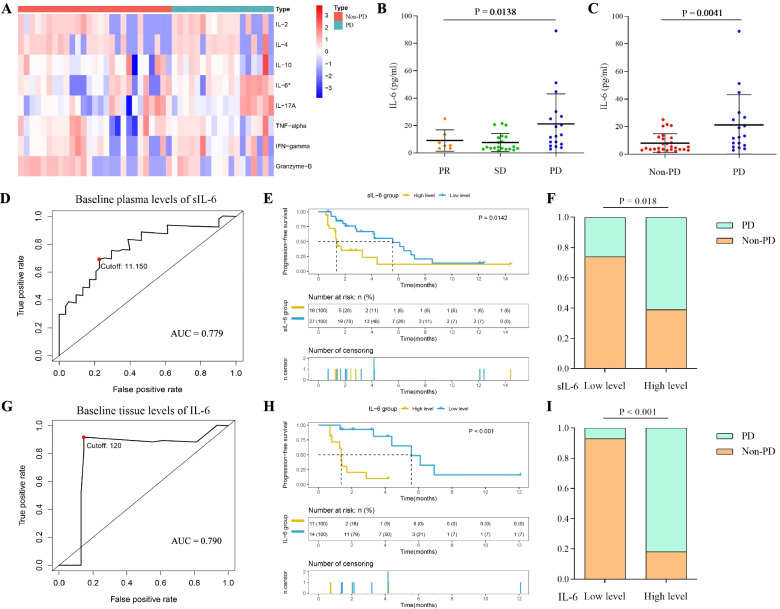
Relationship between baseline plasma or tissue levels of IL-6 and clinical benefits of NSCLC patients after PD-1 inhibitors. **A** Baseline plasma concentration of circulating cytokines between patients with and without PD (PD and non-PD, respectively). **B** The distribution of baseline plasma IL-6 levels among patients exhibiting a PR, SD, and PD. **C** The distribution of baseline plasma IL-6 levels between the PD and non-PD patient cohorts. **D**, **G** ROC analysis of baseline plasma (**D**) and tissue (**G**) IL-6 levels for PFS of patients with NSCLC receiving ICIs in the CICAMS cohort. **E**, **H** Kaplan-Meier survival curve of PFS of patients with NSCLC receiving ICIs based on baseline plasma (**E**) and tissue (**H**) IL-6 levels. **F**, **I** Boxplots evaluating tumor response of NSCLC patients after PD-1 inhibitors between low and high plasma (**F**) and tissue (**I**) levels of IL-6 at baseline. sIL6: secreted IL-6

At the time of the multi-analyte flow assay analysis, the baseline plasma IL-6 concentration significantly differed between patients with the best tumor response as PD and non-PD in the CICAMS cohort, although seven other cytokines were not found to be significantly altered (Fig. 1A). This finding was further evaluated using an ELISA on baseline plasma and tumor tissue levels of IL-6. Consistently, higher baseline levels of IL-6 were observed in patients with PD (plasma sample: Fig. 1B and C; tumor tissue: Additional file 2: Fig. S2). In addition, the potential predictive value of baseline concentration of IL-6 in plasma and tumor tissues for PFS was confirmed with areas under the curve (AUCs) of 0.779 and 0.790, respectively (Fig. 1D and G). According to the best cutoff values calculated from the ROC for PFS, we then stratified patients into the low-level group and high-level group (plasma sample: cutoff = 11.150, Fig. 1D; tumor tissue: cutoff = 120, Fig. 1G). The higher levels of IL-6 protein in blood and tumor tissues were significantly correlated with worse PFS (plasma sample: *P* = 0.0142, Fig. 1E; tumor tissue: *P* < 0.001, Fig. 1H). We also found that patients with PD were observed in high-level group of IL-6 (plasma sample: *P* = 0.018, Fig. 1F; tumor tissue: *P* < 0.001, Fig. 1I). In addition, as an independent and negative predictive factor, IL-6 levels were determined during the univariate and multivariate analyses (plasma sample: Additional file 1: Table S2; tumor tissue: Additional file 1: Table S3). A similar trend was found both in the subgroups of LUSC and LUAD cohorts (plasma sample: Additional file 2: Fig. S1; tumor tissue: Additional file 2: Fig. S3).

### PD-L1 expression is induced by IL-6 via the JAK1/Stat3 signaling pathway

**Fig. 2:**
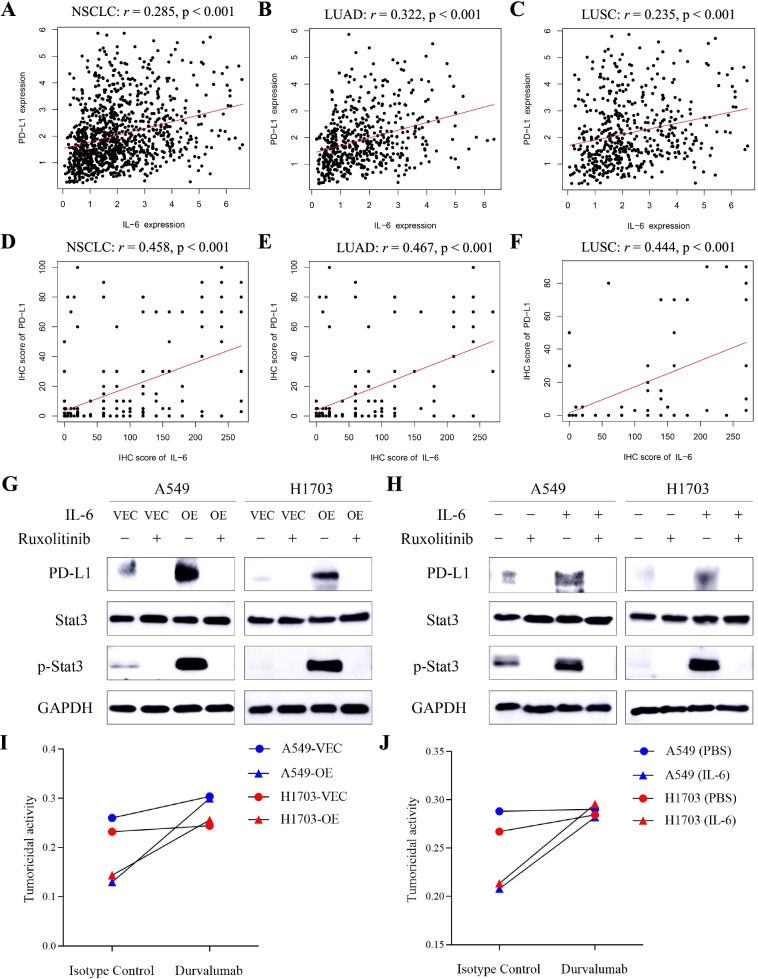
PD-L1 expression is induced by IL-6 via the JAK1/Stat3 signaling pathway. **A**–**C** RNA analysis of the correlation between IL-6 and PD-L1 expression in tumor tissues of patients with NSCLC (**A**), patients with LUAD (**B**), and patients with LUSC (**C**) in the TCGA dataset. **D**–**F** IHC analysis of the correlation between IL-6 and PD-L1 expression in tumor tissues of patients with NSCLC (**D**), patients with LUAD (**E**), and patients with LUSC (**F**) at the CICAMS. **G** Western blotting analysis of PD-L1, Stat3 and p-Stat3 expression in control or IL-6-overexpressimg A549 and H1703 cells with or without cotreatment of ruxolitinib (10umol/L) for 24 h. **H** Western blotting analysis of PD-L1, Stat3 and p-Stat3 expression in A549 and H1703 cells with or without exposure to IL-6 (20 ng/mL) and ruxolitinib (10umol/L) for 24 h. **I** Tumoricidal activity of CD8^+^ T cells cocultured with control or IL-6-overexpressing A549 and H1703 cells after treatment with isotype control and durvalumab (1500 μg/mL) for 48 h. **J** Tumoricidal activity of CD8^+^ T cells cocultured with A549 and H1703 cells in the presence or absence of IL-6 (20 ng/mL) after treatment with isotype control and durvalumab (1,500 μg/mL) for 48 h. VEC, control group; OE, overexpressing group

Based on transcriptome data of 1011 patients with NSCLC (510 patients with LUAD and 501 patients with LUSC) from the TCGA database, a significant positive correlation in mRNA levels between IL-6 and PD-L1 was firstly found (Fig. 2A–C). This correlation was further validated in tumor samples resected from 196 patients with NSCLC (137 patients with LUAD and 59 patients with LUSC) in the CICAMS cohort (Fig. 2D–F). Of note, the results of western blotting analyses showed that cells with high levels of IL-6 tended to have high expression levels of PD-L1 (Additional file 2: Fig. S4). Previous studies demonstrated that IL-6 production is able to activate Janus kinase 1 (JAK1), recruiting and activating the Signal Transducer and Activator of Transcription 3 (Stat3) pathway [27]. The JAK1/Stat3 cascade is a pivotal regulator of immune responses and oncogenesis [28]. Hence, we speculated that IL-6 might induce changes in the expression of PD-L1 in tumors via the JAK1/Stat3 signaling pathway. The western blotting analysis revealed that both IL-6 overexpression and stimulation with exogenous IL-6 protein promoted the phosphorylation of JAK1 and Stat3 and upregulated PD-L1 expression (Additional file 2: Fig. S5). However, PD-L1 expression was decreased after treatment with JAK1/2 inhibitor (ruxolitinib), which blocked the activation of JAK1/Stat3 pathway (Fig. 2G, H). Therefore, we concluded that IL-6 upregulated expression of PD-L1 in tumor via the JAK1/Stat3 signaling pathway.

In addition, the co-culture of CD8^+^ T cells and NSCLC cell lines with IL-6, whether via endogenous overexpression or exogenous exposure, resulted in reduced tumoricidal activity. Intriguingly, durvalumab, an anti-PD-L1 inhibitor, noticeably augmented the tumor-killing ability of CD8^+^ T cells after exposure to IL-6 (Fig. 2I, J). In summary, IL-6 serves as a critical factor involved in the immunosuppression of T cells and tumor cells mediated via the IL-6/JAK1/Stat3 signaling pathway. Further, anti-PD-L1 inhibitors might counteract such immunosuppression.

### Correlation between IL-6 expression and the TIME based on PD-L1 status and CD8^+^ T cell infiltration in patients with NSCLC

**Fig. 3:**
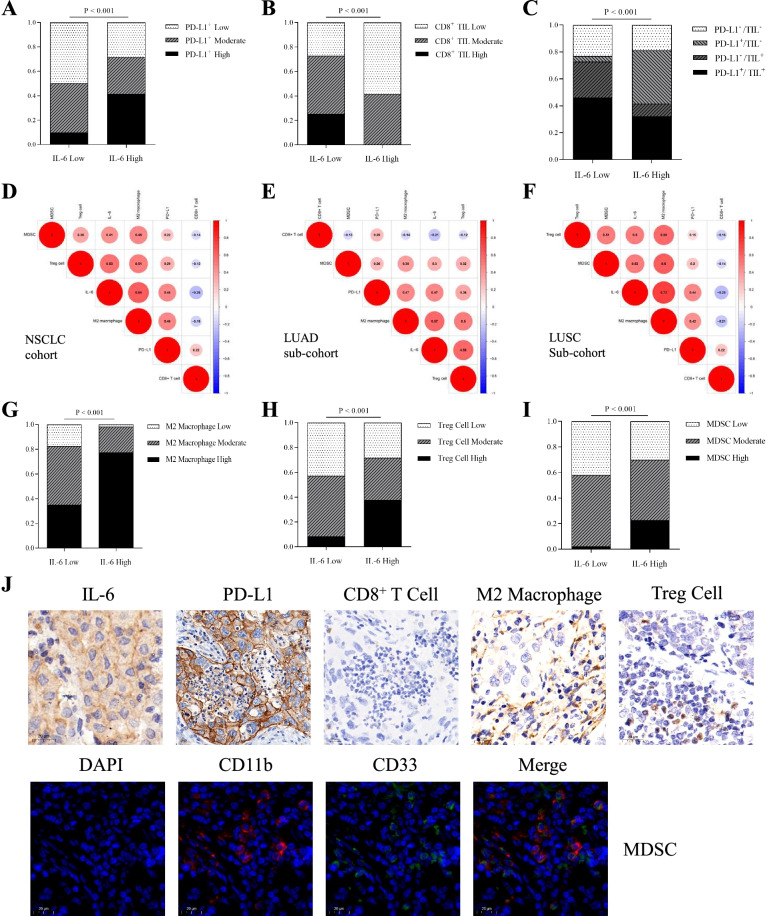
Relationship among IL-6 expression, PD-L1 expression, and immune cell infiltration in patients with NSCLC. **A**, **B** IHC analysis of PD-L1 expression (**A**) and CD8^+^ TIL infiltration (**B**) according to IL-6 expression levels in tumor tissues of patients with NSCLC. **C** IHC analysis of the TIME based on PD-L1 expression and CD8^+^ TIL infiltration according to IL-6 expression levels in tumor tissues of patients with NSCLC. **D**–**F** Correlogram based on Pearson’s *r* values of IL-6 expression, PD-L1 expression, and four tumor-infiltrated immune cell types in tumor tissues of patients with NSCLC (**D**), patients with LUAD (**E**), and patients with LUSC (**F**). **G**–**I** IHC analysis of M2 macrophages (**G**), Treg cells (**H**), and MDSCs (**I**) according to IL-6 expression levels in tumor tissues of patients with NSCLC. **J** Representative IHC images show the correlation of IL-6 expression, PD-L1 expression, and four tumor-infiltrated immune cell types in NSCLC samples. Scale bar = 20 μm

Considering the important role of TIME in immunotherapy, we explored the correlation between IL-6 expression and the TIME in tumor samples with NSCLC from the CICAMS cohort. The results showed that a higher percentage of patients with elevated PD-L1 levels also had high IL-6 levels compared to patients with low levels of PD-L1 (*P* < 0.001, Fig. 3A), indicating that IL-6 positively interacts with the expression of PD-L1. This finding was consistent in patients with LUAD and LUSC (Additional file 2: Fig. S6A and C). As CD8^+^ tumor-infiltrating lymphocytes (TILs) influence the effects of ICIs, we further explored the correlation between IL-6 and CD8^+^ TILs. Our results confirmed that a high level of IL-6 was associated with low infiltrations of CD8^+^ T cells (*P* < 0.001, Fig. 3B), which was also found in patients with LUAD and LUSC (Additional file 2: Fig. S6B and E).

Tumors were classified into four types in this study, based on the PD-L1 status and infiltration of CD8^+^ T cells, as proposed previously [29, 30]. A high percentage of PD-L1^+^/TIL^–^ tumors were found in the high IL-6 level cohort, suggesting that IL-6 might induce an inflammatory phenotype with intrinsic pathway (*P* < 0.001, Fig. 3C). The same phenomenon was observed in patients with LUAD and in patients with LUSC (Additional file 2: Fig. S6C and F).

### Correlation between IL-6 expression and infiltration of immune cells in patients with NSCLC

A correlogram was used to present a positive relationship not only existed between IL-6 and PD-L1 levels, but also with the infiltration of immune cells, including M2 macrophages, MDSCs, and Treg cells in the CICAMS cohort (Fig. 3D–F and Additional file 2: Fig. S7). IHC results revealed that high levels of IL-6 were significantly related with a high percentage of M2 macrophages (*P* < 0.001, Fig. 3G), Treg cells (*P* < 0.001, Fig. 3H), and MDSCs (*P* < 0.001, Fig. 3I). Representative IHC images showed that high levels of IL-6 in tumor samples remarkably correlated with PD-L1 expression and infiltration of M2 macrophages, MDSCs, and Treg cells, but negatively associated with infiltration of CD8^+^ T cells (Fig. 3J). Similar results were found but were not influenced by different pathologies (Additional file 2: Fig. S8). Based on these findings, we hypothesize that IL-6 expression mediates these regulatory factors to promote immunosuppression.

### Mouse models demonstrate that anti-IL-6 combined with anti-PD-L1 antibodies have synergistic antitumor efficacy

**Fig. 4:**
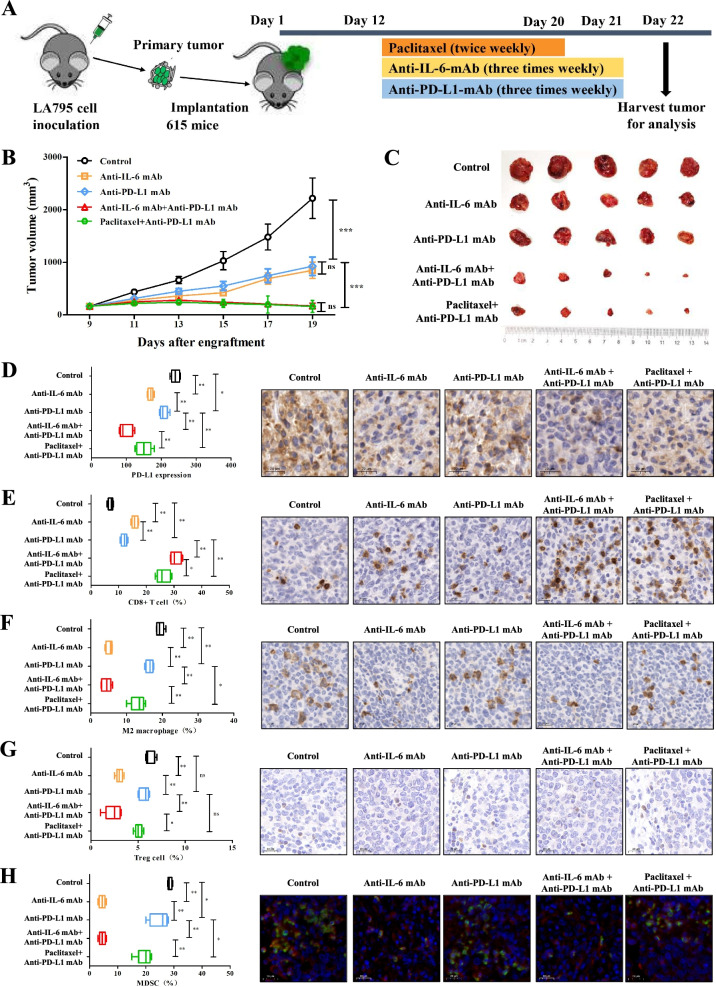
Combined blockade of IL-6 and PD-L1 elicits synergistic antitumor immune responses in a murine model of LUAD. **A** Schemas for constructing the murine model of LUAD and dosing schedule. **B** Mice bearing LA795 cells (*n* = 5) were treated with paclitaxel, anti-IL-6 mAb, and anti-PD-L1 mAb, according to the dosing schedule. Control mice were treated with isotype control. Anti-PD-L1 mAb combined with anti-IL-6 mAb or paclitaxel significantly reduced tumor growth in mice with lung adenocarcinomas. **C** Representative illustrations of tumor nodules in each treated group. **D**–**H** The distributions and representative IHC images of PD-L1 expression (**D**), CD8^+^ T cells (**E**), M2 macrophages (**F**), Treg cells (**G**), and MDSCs (**H**) in each treated group. Scale bar = 20 μm. ns: *P* > 0.05; *P < 0.05; **P < 0.01; ***P < 0.001

**Fig. 5:**
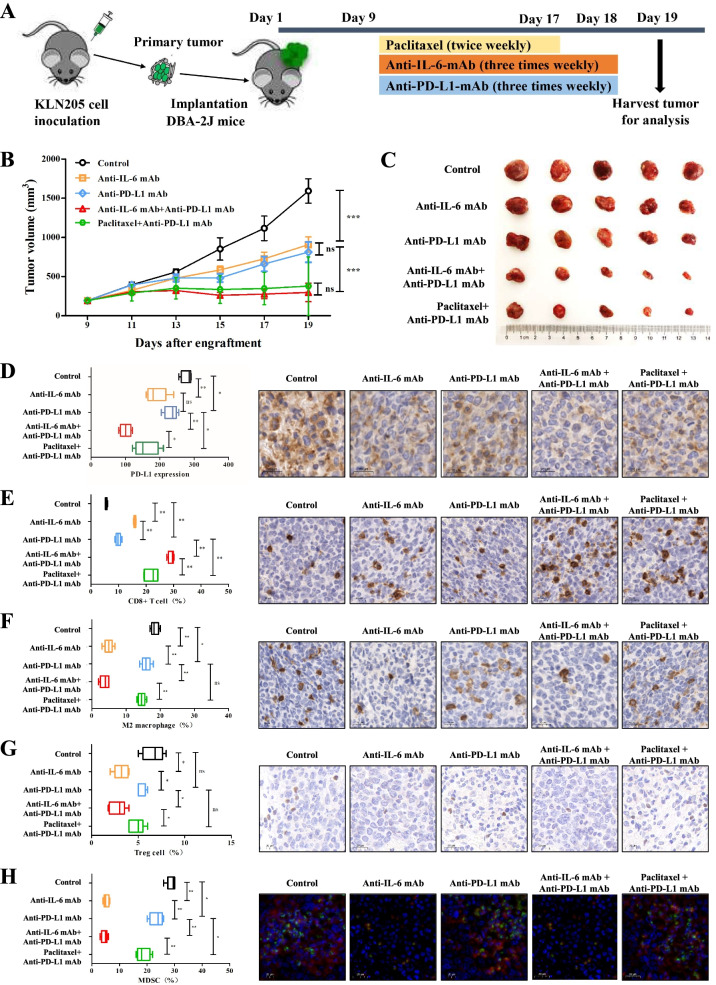
Combined blockade of IL-6 and PD-L1 elicits synergistic antitumor immune responses in a murine model of LUSC. **A** Schemas for constructing the murine model of LUSC and dosing schedule. **B** Mice bearing KLN205 cells (*n* = 5) were treated with paclitaxel, anti-IL-6 mAb, and anti-PD-L1 mAb, according to the dosing schedule. Control mice were treated with isotype control. Anti-PD-L1 mAb combined with anti-IL-6 mAb or paclitaxel significantly reduced tumor growth in mice with lung adenocarcinomas. **C** Representative illustrations of tumor nodules in each treated group. **D**–**H** The distributions and representative IHC images of PD-L1 expression (**D**), CD8^+^ T cells (**E**), M2 macrophages (**F**), Treg cells (**G**), and MDSCs (**H**) in each treated group. Scale bar = 20 μm. ns: *P* > 0.05; *P < 0.05; **P < 0.01; ***P < 0.001

Recognizing that IL-6 expression negatively impacted ICIs therapy, we hypothesized that an anti-IL-6 inhibitor might promote antitumor ability. We tested the efficacy of anti-IL-6 antibodies in murine models of lung cancer (Figs. 4A and 5A). Compared to vehicle control, anti-IL-6 monotherapy markedly suppressed tumors in mouse models. Anti-IL-6 antibodies further enhanced the antitumor ability of anti-PD-L1 inhibition. The synergistic antitumor effects of anti-IL-6 drugs combined with anti-PD-L1 inhibition were similar with the anti-PD-L1 drugs and paclitaxel (Figs. 4B, C and 5B, C). IHC results illustrated that anti-IL-6 antibodies could significantly downregulate PD-L1 expression in tumor cells and decrease the infiltration of M2 macrophages, MDSCs, and Treg cells. Conversely, anti-IL-6 blockade remarkably upregulated the infiltration of CD8^+^ T cells. Intriguingly, compared to PD-L1 inhibitors plus paclitaxel, a common therapeutic strategy used in clinical practice, the noticeable improvement in CD8^+^ T cell, decreased PD-L1 expression, and decreased infiltration of M2 macrophages, MDSCs, and Treg cells were found after IL-6 blockade when the treatment was combined with anti-PD-L1 drugs (Figs. 4D–H and 5D–H). These preclinical data might pave the way for the use of a combined targeting therapy of IL-6 and PD-L1 as a rational, promising therapy to ameliorate immunosuppression state and promote CD8^+^ T cell infiltration.

## Discussion

ICIs targeting PD-1/PD-L1 are characterized by persistent responses and improved survival in patients with advanced NSCLC. Unfortunately, only certain patients can benefit from immunotherapy. Given the imperfect role of PD-L1 expression in tumor specimens in predicting the anti-tumor efficacy of ICIs, effective blood-based biomarkers are urgently needed. Therefore, we used a multi-analyte flow assay to investigate the correlation between baseline plasma concentration of eight cytokines and clinical response in patients with NSCLC treated with ICIs and determined IL-6 to be the most promising predictive cytokine biomarker.

In this study, we demonstrated that the level of IL-6 as a potentially negative predictive biomarker for the outcomes of advanced NSCLC received ICIs. PD-L1 expression was upregulated by IL-6 via the JAK1-Stat3 signaling pathway and simultaneously mediates the PD-L1_high_/TIL_low_ phenotype governed by a low infiltration of CD8^+^ T cells and high numbers of MDSCs, M2 macrophages, and Treg cells. The combined inhibitor of IL-6 and PD-L1 ameliorated the immunosuppressive environment and elicited antitumor efficacy in preclinical animal models, which paves the way for the use of ICIs combined with cytokine-directed therapy.

As a pleiotropic cytokine, IL-6 does not only play a vital role in the response to injury or infection, but also is involved in immune responses, inflammation, and hematopoiesis [32]. It is reported that high expression of IL-6 hads been seen in many types of cancer and it functioned as a poor prognostic factor [32–39]. Consistent with the results of our study, Kang et al. reported that baseline serum IL-6 levels played a negative role in predicting the response to ICIs in NSCLC [17], although there was a difference in the sample collection methods. Additionally, Keegan et al. recently reported that pre-treatment IL-6 levels were not associated with survival when they divided the pretreatment IL-6 expression of 47 patients into four categories and demonstrated that an increase in plasma IL-6 levels correlated with poor PFS in patients with NSCLC received PD-1 inhibitors [40]. The difference between the two studies might be attributed to different way to separate group and small-scale sample sizes. Notably, the race of patients included in the two studies was different. Furthermore, the negative predictive value of baseline plasma IL-6 expression in response to immunotherapy has been previously reported in patients with melanoma and pancreatic cancer [41, 42]. More importantly, we confirmed that higher baseline levels of IL-6, whether in plasma or tumor tissues, were associated with an inferior response to ICIs in patients with NSCLC. The potential predictive value of baseline concentration of IL-6 in plasma and tumor tissues for PFS was confirmed with AUCs of 0.779 and 0.790, respectively. Patients with PD were also observed in a high-level group of IL-6. Stratification and multivariate analyses further revealed that the baseline level of IL-6 in plasma and tumor tissues was an independent prognostic predictor. Nevertheless, due to the study’s retrospective nature, further validation should be performed in prospective studies.

A previous study reported that activation of the IL-6/JAK1 pathway could drive PD-L1 Y112 phosphorylation and result in the evasion of the immune system by tumor cells. In this study, we further found that IL-6 enhanced PD-L1 expression via modulation of the IL-6-JAK1/Stat3 signaling pathway in cancer cells, which also induced immune evasion [43]. However, in an investigation of the relationship between IL-6 and the TIME, our study found that IL-6 expression was positively linked with PD-L1 expression and negatively correlated with CD8^+^ TILs in tumor tissues of patients with NSCLC. When combined with the two variables, a significantly increased proportion of patients with high levels of IL-6 exhibited the PD-L1_high_/TIL_low_ phenotype. A PD-L1_high_/TIL_low_ phenotype is indicative of intrinsic induction, which is frequently observed in cases of driver gene-mutated NSCLC [26], and was reported to impair responses to ICIs [29, 44]. Together, these findings explain why patients with NSCLC and higher levels of IL-6 are resistant to immunotherapy, even with high levels of PD-L1. Furthermore, prior studies showed that IL-6 production attenuated the infiltration of T-helper cells and hindered antigen-specific CD4^+^ T cells that are cognate helpers for CD8^+^ T cells both in vivo and in vitro [31, 45–49].

In addition, IL-6 production significantly positively correlated with the infiltration of MDSCs, M2 macrophage cells, and Treg cells, which indicate that IL-6 is a critical factor in immunosuppression [31, 45, 50]. Hence, we speculated that the neutralization of IL-6 might ameliorate immunosuppression. The synergistic antitumor efficacy of anti-IL-6 and anti-PD-L1 was observed in the murine model. This synergism was also reported in several studies demonstrating that this combination therapy could increase the number of tumor-reactive CD8^+^ T cells and slow tumor growth in tumor-bearing mouse models [20, 21, 41]. However, while downregulated expression of PD-L1 observed in our study, no change in PD-L1 expression was identified in pancreatic tumors, and increased PD-L1 expression in melanoma cells. These differences might be due to tissue- or cell-type-specific properties. Intriguingly, significantly higher infiltration of CD8^+^ T cells and reduced PD-L1 expression were noticed after the combination blockade of PD-L1 and IL-6. These findings might be explained by the neutralization of IL-6, which has been shown to attenuate immunosuppression and improve effector T cell functional capacity [21, 41].

Although the antitumor efficacy of combination blockade of PD-L1 and IL-6 was similar with anti-PD-L1 drugs and paclitaxel in mice studies, lower PD-L1 expression and lower infiltration of immunosuppressive cells was found in the tumor microenvironment in the anti-PD-L1 drugs combined with anti-IL-6 inhibitors. Based on this phenomenon, we speculated that a combination blockade of PD-L1 and IL-6 might alleviate immunosuppressive states more deeply and lead to longer PFS, which was noteworthy to explore in further study. Additional addition of IL-6 inhibitors would be a better choice for NSCLC patients who were intolerant to chemotherapy. Moreover, many studies reported that significantly elevated expression of plasma IL-6 was detected while immune-related adverse events (irAEs) or even cytokine release syndrome occurred [51–54]. A recent study also revealed that tocilizumab, an IL-6R blockade, was a well-tolerated and effective treatment for both prevention and management of irAEs [55]. These results reminded us that the combined blockade of IL-6 and PD-1/PD-L1, compared to chemotherapy and anti-PD-1/PD-L1, might be a more suitable therapy for NSCLC patients with a high level of IL-6. Delightfully, a combinatorial approach with tocilizumab and PD-1/PD-L1 blockades is under clinical development (NCT04940299, NCT04691817, NCT03337698), including lung cancer.

## Conclusions

Our study elucidated the role of baseline IL-6 levels in predicting treatment outcomes and inducing resistance to immunotherapy in patients with NSCLC. Baseline levels of IL-6 in plasma may emerge as a potential predictive biomarker for immunotherapy and help further optimize the ICIs paradigm of personalized medicine for patients with advanced NSCLC. Meanwhile, our results indicated that the targeted inhibitor of IL-6 may augment the efficacy of anti-PD-L1 in NSCLC, paving the path for the utilization of IL-6 as a therapeutic target for cancer treatment.

## Supplementary Information

**Additional file 1: Table S1.** Demographic characteristics of NSCLC patients treated with anti-PD-1 inhibitors. **Table S2.** Univariate and multivariate regression analyses of the association between baseline plasma IL-6 levels and clinical factors for the prediction of PFS. **Table S3.** Univariate and multivariate regression analyses of the relationship between baseline tumor tissue IL-6 levels and clinical factors for the prediction of PFS.

**Additional file 2: Figure S1.** Relationship between baseline plasma levels of IL-6 and clinical benefits to patients with NSCLC receiving ICIs in the CICAMS cohort. **Figure S2.** Relationship between baseline tumor tissue levels of IL-6 and tumor response of NSCLC patients after PD-1 inhibitors in the CICAMS cohort. **Figure S3.** Relationship between baseline tumor tissue levels of IL-6 and clinical benefit of patients with NSCLC receiving ICIs in the CICAMS cohort. **Figure S4.** Western blotting analysis of PD-L1 and IL-6 expression in normal lung cell and NSCLC cells of humans. **Figure S5.** Correlation among IL-6 expression, PD-L1 expression, and the JAK1/Stat3 signaling pathway. **Figure S6.** Correlation between IL-6 expression and the tumor microenvironment based on PD-L1 and CD8^+^ T cell infiltration in LUAD and LUSC patients. **Figure S7.** Correlograms of IL-6 expression with four tumor-infiltrated immune cells in NSCLC patients. **Figure S8.** Correlation between IL-6 expression and immune cell infiltration in patients with LUAD and LUSC.

## Data Availability

The datasets used and/or analyzed in this study can be obtained from the corresponding authors as reasonably required.

## Abbreviations

NSCLC: Non-small cell lung cancer
ICIs: Immune checkpoint inhibitors
OS: Overall survival
IFNs: Interferons
TIME: Tumor immune microenvironment
CR: Complete response
PR: Partial response
SD: Stable disease
PD: Progressive disease
PFS: Progression-free survival
ELISA: Enzyme-linked immunosorbent assay
FFPE: Formalin fixation paraffin embedding
LUAD: Lung adenocarcinoma
LUSC: Lung squamous carcinoma
PBMCs: Peripheral blood mononuclear cells
AUC: Areas under the curve
irAEs: Immune-related adverse events
