# Correction: Systematic analysis of IL-6 as a predictive biomarker and desensitizer of immunotherapy responses in patients with non-small cell lung cancer

**DOI:** 10.1186/s12916-022-02492-0

**Published:** 2022-07-26

**Authors:** Chengming Liu, Lu Yang, Haiyan Xu, Sufei Zheng, Zhanyu Wang, Sihui Wang, Yaning Yang, Shuyang Zhang, Xiaoli Feng, Nan Sun, Yan Wang, Jie He

**Affiliations:** 1 Department of Thoracic Surgery, National Cancer Center/National Clinical Research Center for Cancer/Cancer Hospital, Chinese Academy of Medical Sciences and Peking Union Medical College, Beijing, 100021 China; 2 State Key Laboratory of Molecular Oncology, National Cancer Center/National Clinical Research Center for Cancer/Cancer Hospital, Chinese Academy of Medical Sciences and Peking Union Medical College, Beijing, 100021 China; 3 Department of Medical Oncology and Radiation Sickness, Peking University Third Hospital, Beijing, 100191 China; 4 Department of Medical Oncology, National Cancer Center/National Clinical Research Center for Cancer/Cancer Hospital, Chinese Academy of Medical Sciences and Peking Union Medical College, Beijing, 100021 China; 5 Department of Comprehensive Oncology, National Cancer Center/ National Clinical Research Center for Cancer/Cancer Hospital, Chinese Academy of Medical Sciences and Peking Union Medical College, Beijing, 100021 China; 6 Department of Pathology, National Cancer Center/National Clinical Research Center for Cancer/Cancer Hospital, Chinese Academy of Medical Sciences and Peking Union Medical College, Beijing, 100021 China

**Correction: BMC Med 20, 187 (2022)**

**https://doi.org/10.1186/s12916-022-02356-7**

After publication of the original article [1], it came to the authors’ attention that there is an error in the originally-published version of **Additional file** **1****: Table S2 and Table S3**. In the univariate and multivariate regression analyses of the association between baseline plasma or tumor tissue IL-6 levels and clinical factors for the prediction of progression-free survival (PFS), the results of smoking history were lost.

The correct version of **Additional file** **1****: Table S2 and Table S3** is published in this erratum.

## Supplementary Information

**Additional file 1: Table S2**. Univariate and multivariate regression analyses of the association between baseline plasma IL-6 levels and clinical factors for the prediction of PFS. **Table S3.** Univariate and multivariate regression analyses of the relationship between baseline tumor tissue IL-6 levels and clinical factors for the prediction of PFS.
