# Supplementary material for: Systematic analysis of IL-6 as a predictive biomarker and desensitizer of immunotherapy responses in patients with non-small cell lung cancer
Source: BMC Med. 2022 May 13;20:187. doi: 10.1186/s12916-022-02356-7 (PMC9102328; doi:10.1186/s12916-022-02356-7)
Supplement: Supplementary file 1 — Additional file 1: Table S1. Demographic characteristics of NSCLC patients treated with anti-PD-1 inhibitors. Table S2. Univariate and multivariate regression analyses of the association between baseline plasma IL-6 levels and clinical factors for the prediction of PFS. Table S3. Univariate and multivariate regression analyses of the relationship between baseline tumor tissue IL-6 levels and clinical factors for the prediction of PFS. [file 12916_2022_2356_MOESM1_ESM.docx]

**Table S1.** Demographic characteristics of NSCLC patients treated with anti-PD-1 inhibitors.

| Characteristics | Population (sIL-6, ELISA) | Population (IL-6, IHC) |
| --- | --- | --- |
| Case No.  Gender, n (%) | 45 | 25 |
| Male | 35 (77.8) | 18 (72) |
| Female | 10 (22.2) | 7 (15.6) |
| Age, n (%) |  |  |
| ＜60 | 21 (46.7) | 12 (26.7) |
| ≥60 | 24 (53.3) | 13 (28.9) |
| Smoking history, n (%) |  |  |
| Never | 14 (31.1) | 9 (20) |
| Ever | 31 (68.9） | 16 (35.6） |
| Pathology, n (%) |  |  |
| LUAD | 20 (44.4) | 8 (17.8) |
| LUSC | 25 (55.6） | 17 (37.8） |
| Staging, n (%) |  |  |
| III | 5 (11.1) | 3 (6.7) |
| IV | 40 (88.9) | 22 (48.9) |
| Mutation status, n (%) |  |  |
| Wild type | 1 (2.2) | 0 (0) |
| EGFR mutation/ALK fusion | 10 (22.2) | 7 (15.6) |
| Other mutation | 8 (17.8) | 2 (4.4) |
| Unknown | 26 (57.8) | 16 (35.6) |
| ECOG, n (%) |  |  |
| 0 | 1 (2.2） | 1 (2.2） |
| 1 | 39 (86.7) | 21 (46.7) |
| 2 | 5 (11.1) | 3 (6.7) |
| Therapy lines, n (%) |  |  |
| 1 | 7 (15.6） | 2 (4.4） |
| 2 | 20 (44.4) | 13 (28.9) |
| ≥3 | 18 (40.0) | 10 (22.2) |
| Study drugs, n (%) |  |  |
| Nivolumab | 2 (4.4) | 0 (0) |
| Pembrolizumab | 23 (51.1) | 15 (33.3) |
| Sintilimab | 19 (42.2) | 10 (22.2) |
| Toripalimab | 1 (2.2) | 0 (0) |
| Toxicity, n (%) |  |  |
| Yes | 13 (28.9） | 7 (15.6） |
| No | 32 (71.1) | 18 (40.0) |
| Antibiotic history, n (%) |  |  |
| Yes | 5 (11.1） | 2 (4.4） |
| No | 40 (88.8) | 23 (51.1) |
| Response evaluation, n (%) |  |  |
| PR | 7 (15.6） | 2 (8.0） |
| SD | 20 (44.4） | 13 (52.0） |
| PD | 18 (40.0) | 10 (40.0) |

**Table S2.** Univariate and multivariate regression analyses of the association between baseline plasma IL-6 levels and clinical factors for the prediction of PFS.

|  | Univariable analysis | | Multivariable analysis | |
| --- | --- | --- | --- | --- |
|  | P-value | HR（95%CI） | P-value | HR（95%CI） |
| Age | 0.687 | 0.991（0.949-1.035） |  |  |
| Gender | 0.446 | 0.711（0.296-1.709） |  |  |
| Pathology | 0.022 | 0.414（0.195-0.880） | 0.229 | 0.543（0.201-1.469） |
| Staging | 0.865 | 1.110（0.333-3.699） |  |  |
| Mutation status | 0.047 | 1.346（1.004-1.805） | 0.051 | 1.464（0.999-2.146） |
| ECOG PS | 0.185 | 0.511（0.190-1.378） |  |  |
| Therapy lines | 0.023 | 1.246（1.031-1.507） | 0.158 | 1.170（0.941-1.456） |
| Study drug | 0.252 | 0.741（0.443-1.238） |  |  |
| Toxicity | 0.074 | 0.437（0.177-1.082） | 0.080 | 0.426（0.164-1.106） |
| Antibiotic history | 0.582 | 1.350（0.464-3.931） |  |  |
| **sIL-6 level** | **＜0.001** | 1.053（1.025-1.081） | **＜0.001** | 1.079（1.044-1.115） |

**Table S3.** Univariate and multivariate regression analyses of the relationship between baseline tumor tissue IL-6 levels and clinical factors for the prediction of PFS.

|  | Univariable analysis | | Multivariable analysis | |
| --- | --- | --- | --- | --- |
|  | P-value | HR（95%CI） | P-value | HR（95%CI） |
| Age | 0.200 | 0.967（0.919-1.018） |  |  |
| Gender | 0.467 | 0.633（0.184-2.173） |  |  |
| Pathology | 0.005 | 0.171（0.049-0.595） | 0.007 | 0.032（0.003-0.394） |
| Staging | 0.024 | 0.202（0.050-0.813） | 0.003 | 0.023（0.002-0.286） |
| Mutation status | 0.340 | 1.249（0.791-1.974） |  |  |
| ECOG PS | 0.844 | 0.879（0.244-3.171） |  |  |
| Therapy lines | 0.007 | 1.709（1.155-2.528） | 0.126 | 1.446（0.901-2.321） |
| Study drug | 0.232 | 0.515（0.173-1.530） |  |  |
| Toxicity | 0.585 | 0.725（0.229-2.295） |  |  |
| Antibiotic history | 0.544 | 1.606（0.348-7.405） |  |  |
| **IL-6 level** | **0.012** | 1.055（0.997-1.094） | **0.017** | 1.059（0.996-1.092） |
