# Supplementary material for: Systematic analysis of IL-6 as a predictive biomarker and desensitizer of immunotherapy responses in patients with non-small cell lung cancer
Source: BMC Med. 2022 May 13;20:187. doi: 10.1186/s12916-022-02356-7 (PMC9102328; doi:10.1186/s12916-022-02356-7)

**Fig. S1. Relationship between baseline plasma levels of IL-6 and clinical benefits to patients with NSCLC receiving ICIs in the CICAMS cohort.** (A and C) ROC analysis of baseline plasma levels of IL-6 for PFS of patients with LUAD (A) and LUSC (C) receiving ICIs in the CICAMS cohort. (B and D) Kaplan-Meier survival curve of PFS of patients with LUAD (B) and LUSC (D) receiving ICIs based on baseline plasma levels of IL-6.


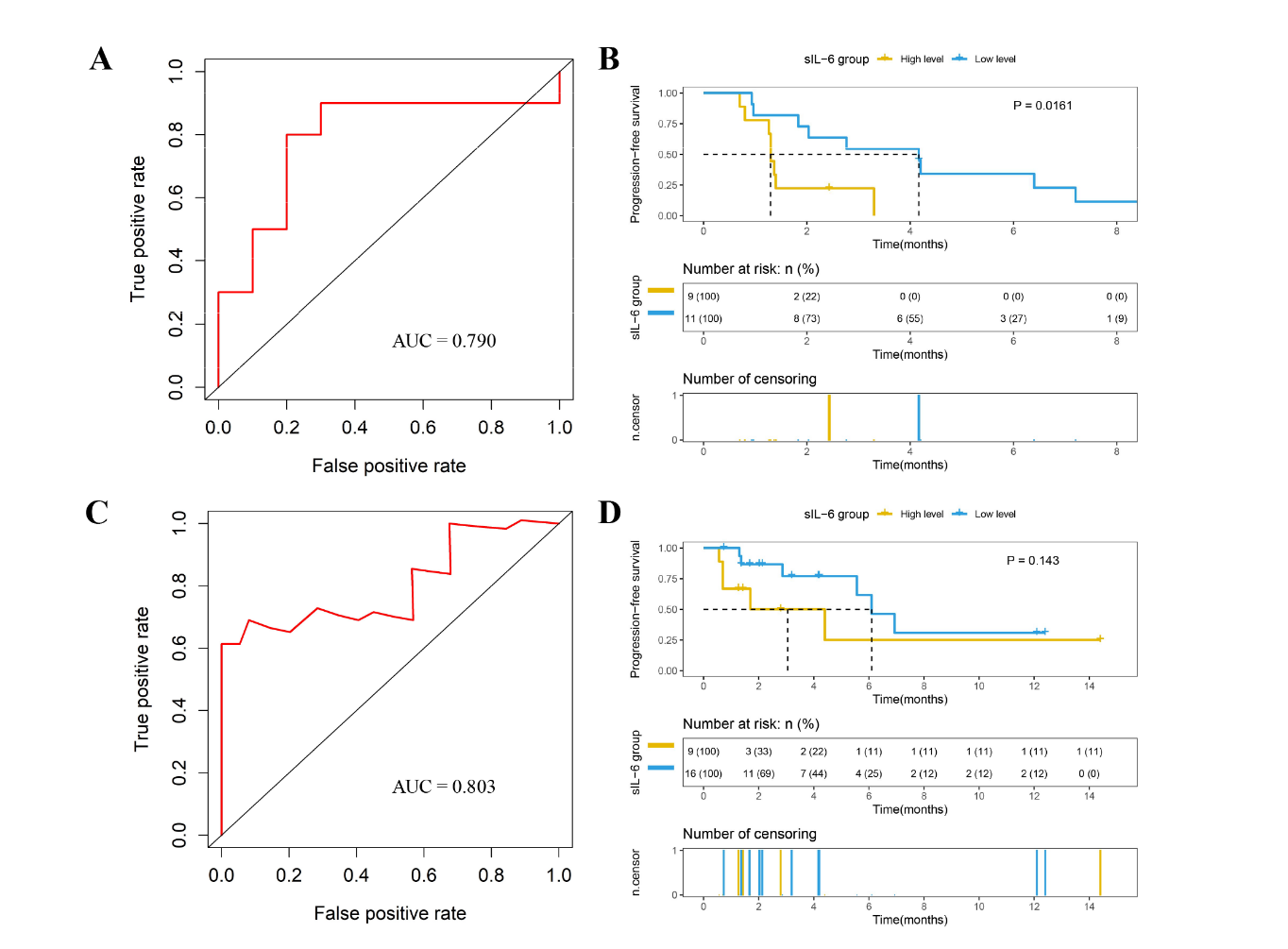


**Fig. S2. Relationship between baseline tumor tissue levels of IL-6 and tumor response of NSCLC patients after PD-1 inhibitors in the CICAMS cohort.** (A) The distribution of baseline tumor tissue IL-6 levels among patients exhibiting a PR, SD, and PD. (B) The distribution of baseline tumor tissue IL-6 levels between patients with and without PD (PD and non-PD, respectively).


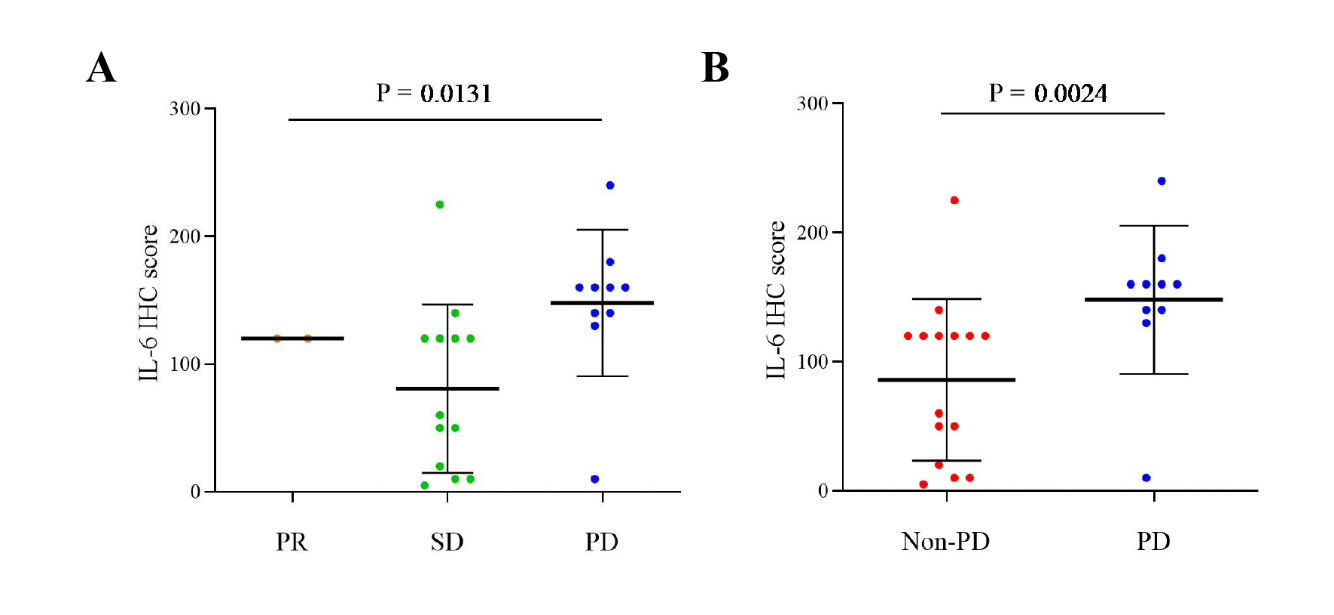


**Fig. S3. Relationship between baseline tumor tissue levels of IL-6 and clinical benefit of patients with NSCLC receiving ICIs in the CICAMS cohort.** (A and C) ROC analysis of baseline tumor tissue levels of IL-6 for PFS of patients with LUAD (A) and LUSC (C) receiving ICIs in the CICAMS cohort. (B and D) Kaplan-Meier survival curve of PFS of patients with LUAD (B) and LUSC (D) receiving ICIs based on baseline tumor tissue levels of IL-6.


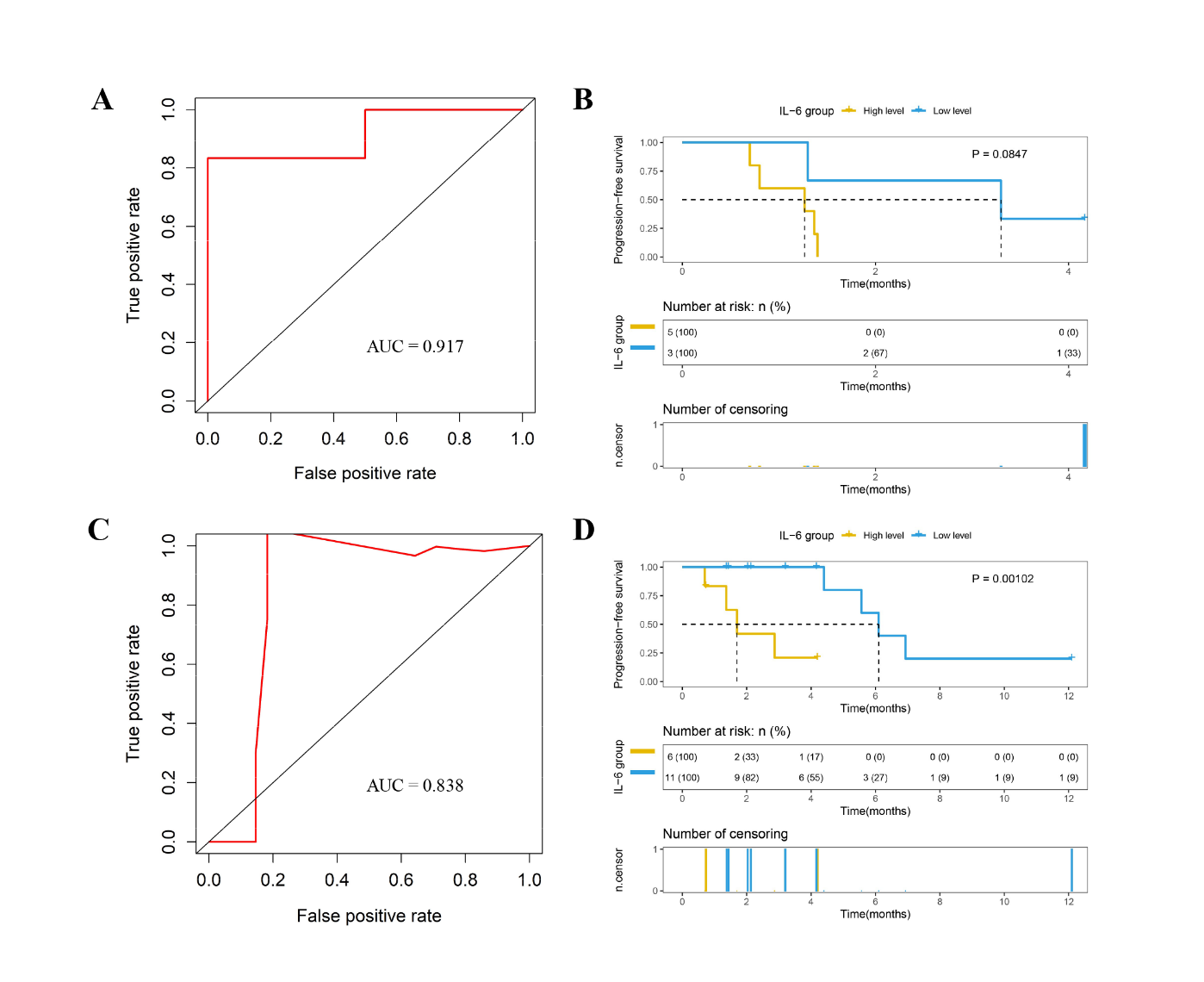


**Fig.** **S4.** **Western blotting analysis of PD-L1 and IL-6 expression in normal lung cell and NSCLC cells of humans.**


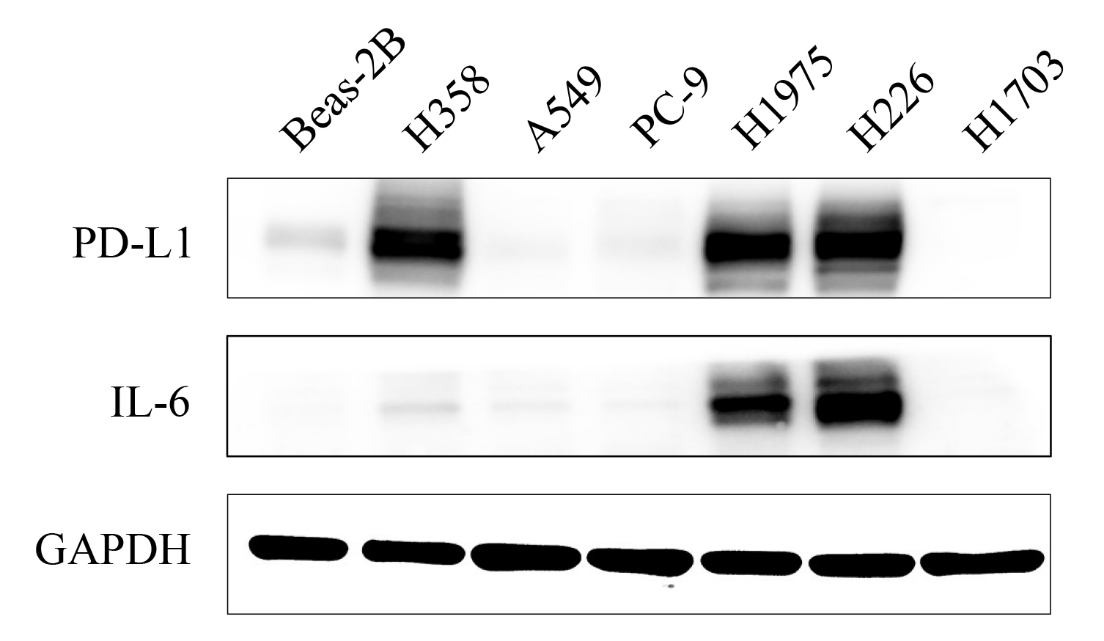


**Fig. S5. Correlation among IL-6 expression, PD-L1 expression and the JAK1/Stat3 signaling pathway.** (A and B) Western blotting analysis of PD-L1, JAK1, p-JAK1, Stat3, and p-Stat3 expression in control or IL-6-overexpressimg A549 (A) and H1703 (B) cells. (C and D) Western blotting analysis of PD-L1, JAK1, and Stat3 expression in A549 (C) and H1703 (D) cells with or without exposure to IL-6 (20 ng/mL). VEC: control group; OE: overexpressing group.


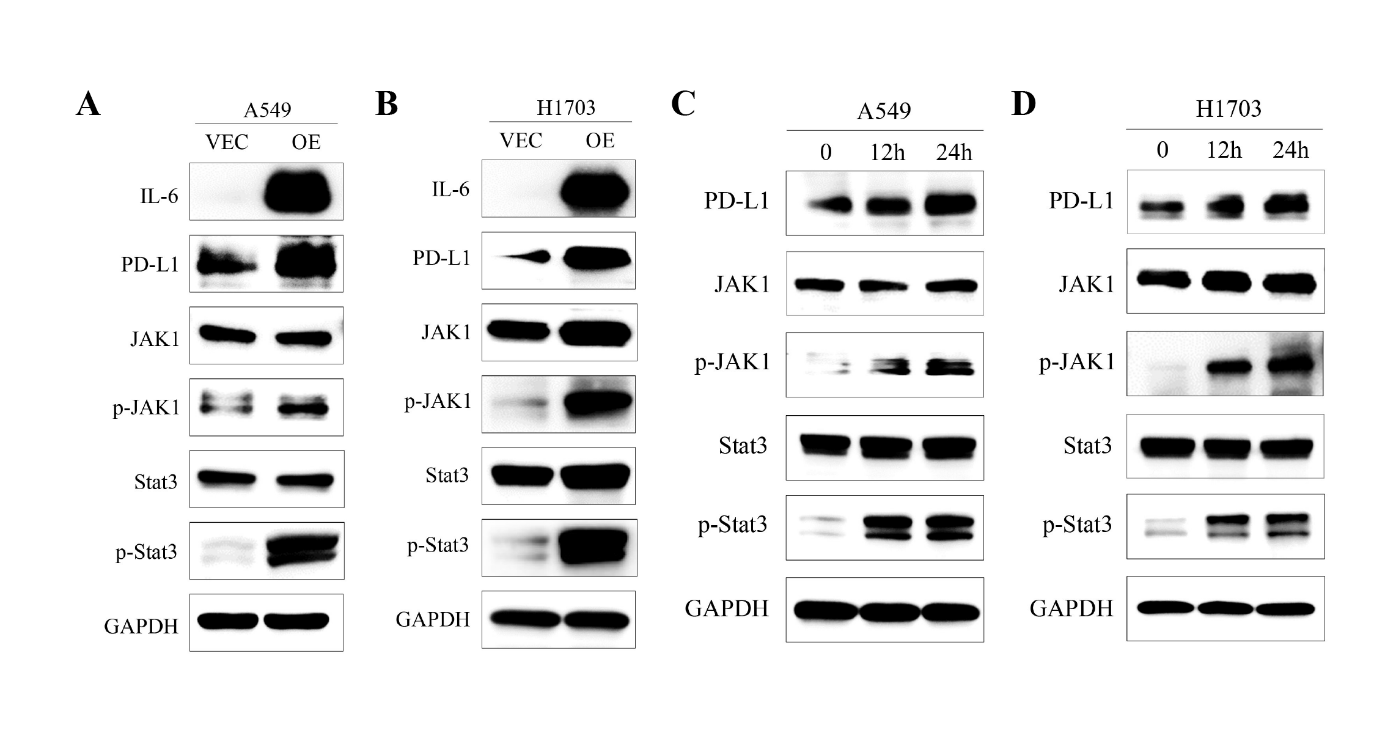


**Fig. S6. Correlation between IL-6 expression and the tumor microenvironment based on PD-L1 and CD8^+^ T cell infiltration in LUAD and LUSC patients.** (A and D) IHC analysis of PD-L1 expression according to IL-6 expression levels in tumor tissues of patients with LUAD (A) and LUSC (D). (B and E) IHC analysis of CD8^+^ T cell infiltration according to IL-6 expression levels in tumor tissues of patients with LUAD (B) and LUSC (E). (C and F) IHC analysis of tumor microenvironment based on PD-L1 and CD8^+^ T cell infiltration according to IL-6 expression levels in tumor tissues of patients with LUAD (C) and LUSC (F).


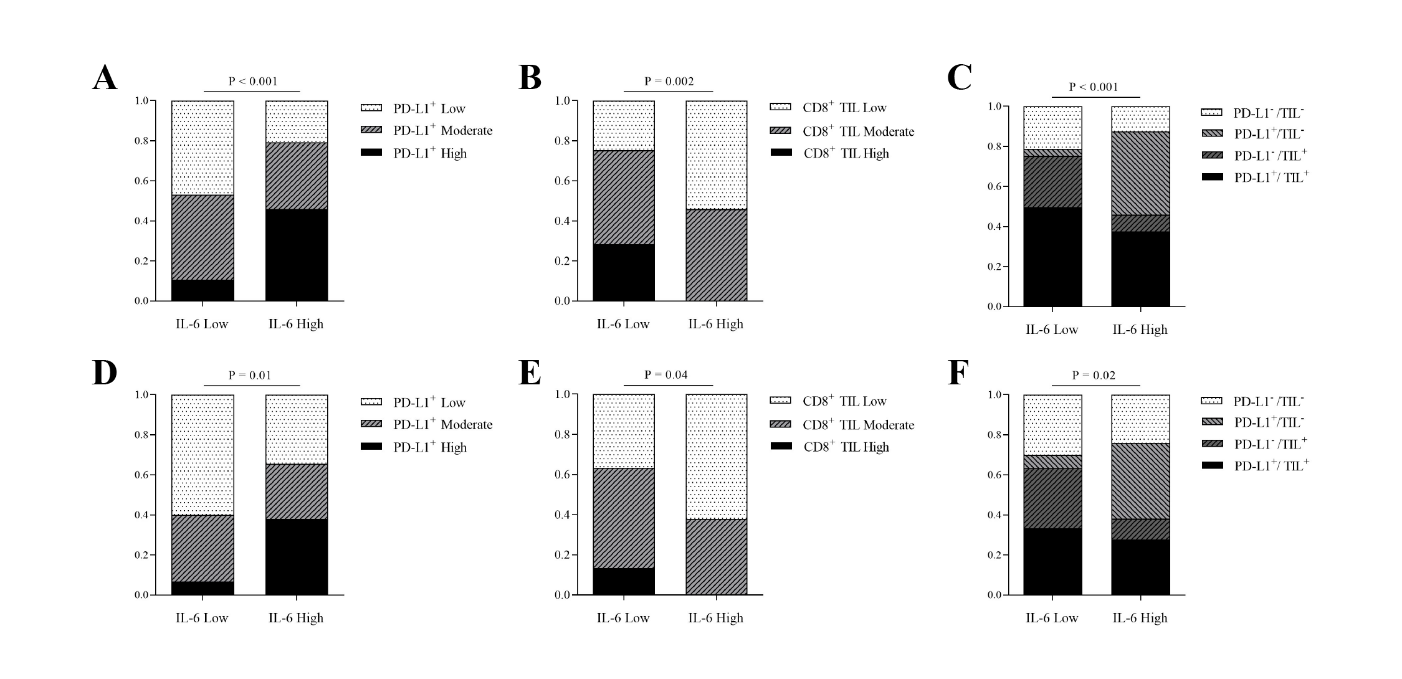


**Fig. S7.** **Correlograms of IL-6 expression with four tumor-infiltrated immune cells in NSCLC patients.** (A, B and C) Correlograms of IL-6 expression with CD8^+^ T cell in tumor tissues of patients with NSCLC (A), LUAD (B) and LUSC (C). (D, E and F) Correlograms of IL-6 expression with M2 macrophage in tumor tissues of patients with NSCLC (D), LUAD (E) and LUSC (F). (G, H and I) Correlograms of IL-6 expression with Treg cell in tumor tissues of patients with NSCLC (G), LUAD (H) and LUSC (I). (J, K and L) Correlograms of IL-6 expression with MDSC in tumor tissues of patients with NSCLC (J), LUAD (K) and LUSC (L).


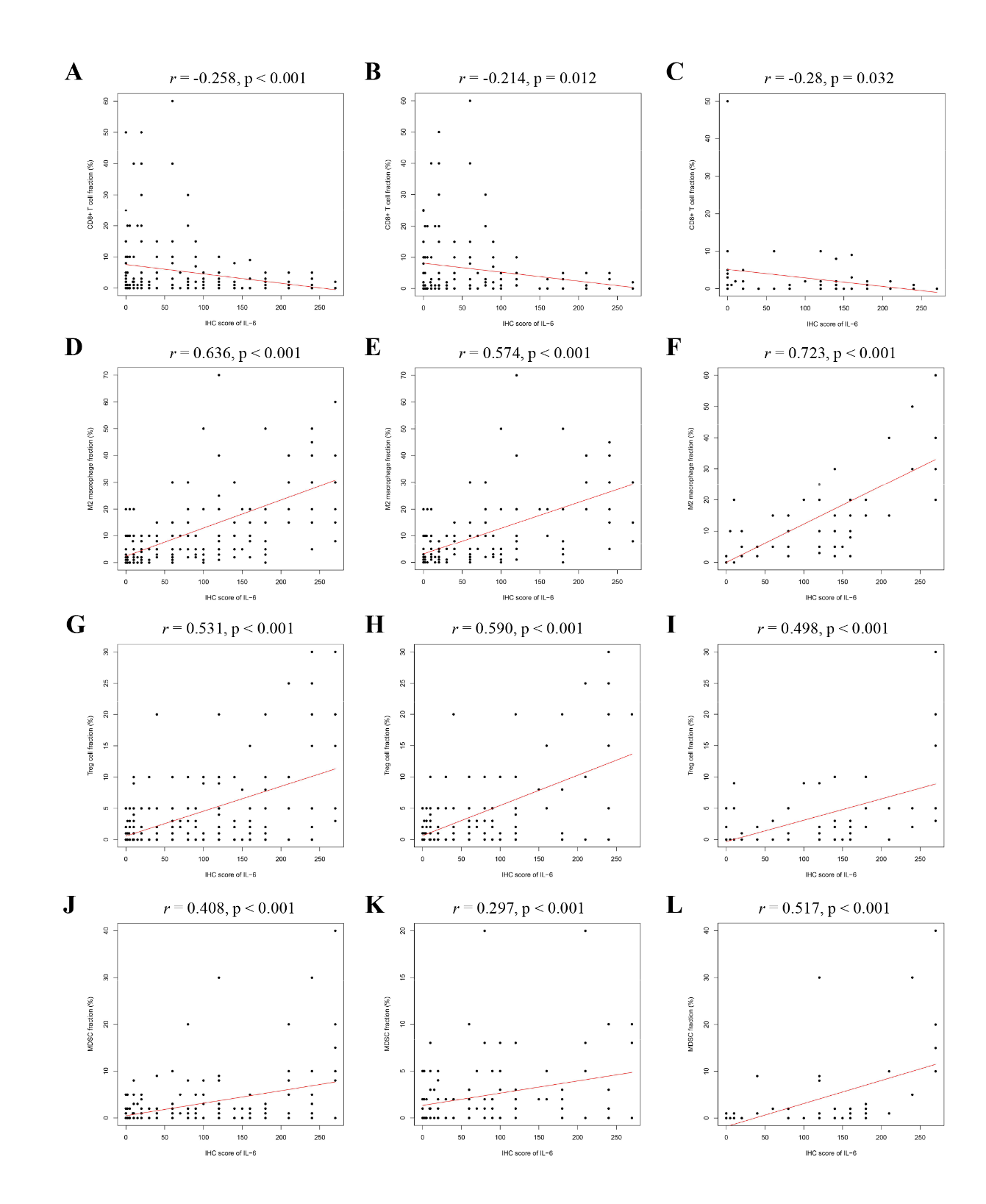


**Fig.** **S8.** **Correlation between IL-6 expression and immune cell infiltration in patients with LUAD and LUSC.** (A and D) IHC analysis of M2 macrophage according to IL-6 expression levels in tumor tissues of patients with LUAD (A) and LUSC (D). (B and E) IHC analysis of Treg cell according to IL-6 expression levels in tumor tissues of patients with LUAD (B) and LUSC (E). (C and F) IHC analysis of MDSC according to IL-6 expression levels in tumor tissues of patients with LUAD (C) and LUSC (F).


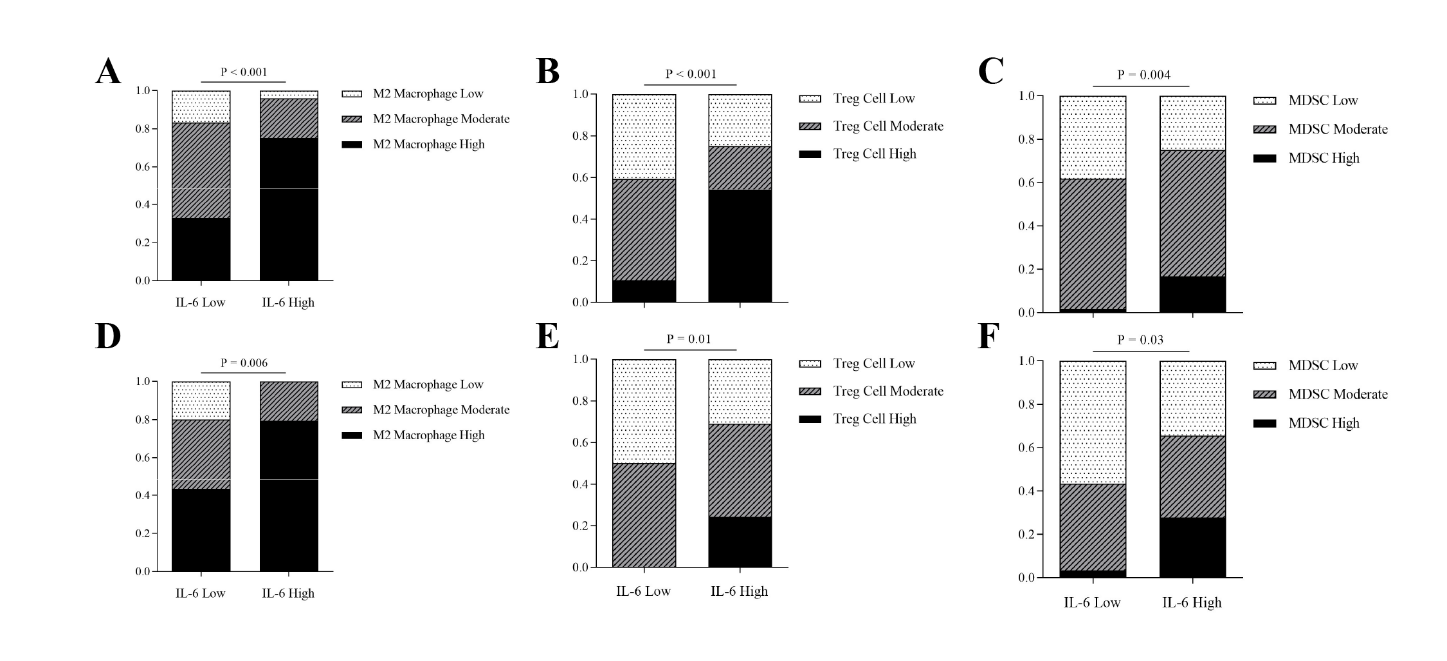

Supplement: Supplementary file 2 — Additional file 2: Figure S1. Relationship between baseline plasma levels of IL-6 and clinical benefits to patients with NSCLC receiving ICIs in the CICAMS cohort. Figure S2. Relationship between baseline tumor tissue levels of IL-6 and tumor response of NSCLC patients after PD-1 inhibitors in the CICAMS cohort. Figure S3. Relationship between baseline tumor tissue levels of IL-6 and clinical benefit of patients with NSCLC receiving ICIs in the CICAMS cohort. Figure S4. Western blotting analysis of PD-L1 and IL-6 expression in normal lung cell and NSCLC cells of humans. Figure S5. Correlation among IL-6 expression, PD-L1 expression, and the JAK1/Stat3 signaling pathway. Figure S6. Correlation between IL-6 expression and the tumor microenvironment based on PD-L1 and CD8+ T cell infiltration in LUAD and LUSC patients. Figure S7. Correlograms of IL-6 expression with four tumor-infiltrated immune cells in NSCLC patients. Figure S8. Correlation between IL-6 expression and immune cell infiltration in patients with LUAD and LUSC. [file 12916_2022_2356_MOESM2_ESM.docx]
