# Supplementary material for: Correction: Systematic analysis of IL-6 as a predictive biomarker and desensitizer of immunotherapy responses in patients with non-small cell lung cancer
Source: BMC Med. 2022 Jul 26;20:265. doi: 10.1186/s12916-022-02492-0 (PMC9327161; doi:10.1186/s12916-022-02492-0)
Supplement: Supplementary file 1 — Additional file 1: Table S2. Univariate and multivariate regression analyses of the association between baseline plasma IL-6 levels and clinical factors for the prediction of PFS. Table S3. Univariate and multivariate regression analyses of the relationship between baseline tumor tissue IL-6 levels and clinical factors for the prediction of PFS. [file 12916_2022_2492_MOESM1_ESM.docx]

**Table S2.** Univariate and multivariate regression analyses of the association between baseline plasma IL-6 levels and clinical factors for the prediction of PFS.

|  | Univariable analysis | | Multivariable analysis | |
| --- | --- | --- | --- | --- |
|  | P-value | HR（95%CI） | P-value | HR（95%CI） |
| Age | 0.687 | 0.991（0.949-1.035） |  |  |
| Gender | 0.446 | 0.711（0.296-1.709） |  |  |
| Smoking history | 0.484 | 0.760（0.352-1.641） |  |  |
| Pathology | 0.022 | 0.414（0.195-0.880） | 0.229 | 0.543（0.201-1.469） |
| Staging | 0.865 | 1.110（0.333-3.699） |  |  |
| Mutation status | 0.047 | 1.346（1.004-1.805） | 0.051 | 1.464（0.999-2.146） |
| ECOG PS | 0.185 | 0.511（0.190-1.378） |  |  |
| Therapy lines | 0.023 | 1.246（1.031-1.507） | 0.158 | 1.170（0.941-1.456） |
| Study drug | 0.252 | 0.741（0.443-1.238） |  |  |
| Toxicity | 0.074 | 0.437（0.177-1.082） | 0.080 | 0.426（0.164-1.106） |
| Antibiotic history | 0.582 | 1.350（0.464-3.931） |  |  |
| **sIL-6 level** | **＜0.001** | 1.053（1.025-1.081） | **＜0.001** | 1.079（1.044-1.115） |

**Table S3.** Univariate and multivariate regression analyses of the relationship between baseline tumor tissue IL-6 levels and clinical factors for the prediction of PFS.

|  | Univariable analysis | | Multivariable analysis | |
| --- | --- | --- | --- | --- |
|  | P-value | HR（95%CI） | P-value | HR（95%CI） |
| Age | 0.200 | 0.967（0.919-1.018） |  |  |
| Gender | 0.467 | 0.633（0.184-2.173） |  |  |
| Smoking history | 0.974 | 0.982（0.332-2.902） |  |  |
| Pathology | 0.005 | 0.171（0.049-0.595） | 0.007 | 0.032（0.003-0.394） |
| Staging | 0.024 | 0.202（0.050-0.813） | 0.003 | 0.023（0.002-0.286） |
| Mutation status | 0.340 | 1.249（0.791-1.974） |  |  |
| ECOG PS | 0.844 | 0.879（0.244-3.171） |  |  |
| Therapy lines | 0.007 | 1.709（1.155-2.528） | 0.126 | 1.446（0.901-2.321） |
| Study drug | 0.232 | 0.515（0.173-1.530） |  |  |
| Toxicity | 0.585 | 0.725（0.229-2.295） |  |  |
| Antibiotic history | 0.544 | 1.606（0.348-7.405） |  |  |
| **IL-6 level** | **0.012** | 1.055（0.997-1.094） | **0.017** | 1.059（0.996-1.092） |
